# Barriers to Completing TB Diagnosis in Yemen: Services Should Respond to Patients' Needs

**DOI:** 10.1371/journal.pone.0105194

**Published:** 2014-09-22

**Authors:** Rachel M. Anderson de Cuevas, Najla Al-Sonboli, Nasher Al-Aghbari, Mohammed A. Yassin, Luis E. Cuevas, Sally J. Theobald

**Affiliations:** 1 Department of International Public Health, Liverpool School of Tropical Medicine, Liverpool, United Kingdom; 2 Medical Faculty, Sana'a University, Sana'a, Yemen; 3 Department of Clinical Sciences, Liverpool School of Tropical Medicine, Liverpool, United Kingdom; Johns Hopkins Bloomberg School of Public Health, United States of America

## Abstract

**Objectives and Background:**

Obtaining a diagnosis of tuberculosis (TB) is a prerequisite for accessing specific treatment, yet one third of estimated new cases are missed worldwide by National Programmes. This study investigated economic, geographical, socio-cultural and health system factors hindering adults' attendance and completion of the TB diagnostic process in Yemen, to inform interventions designed to improve patient access to services.

**Methodology:**

The study employed a mixed methods design comprising a cross-sectional survey and In-Depth-Interviews (IDIs) and Focus Group Discussions (FGDs) among patients abandoning the diagnosis or registering for treatment. Adults with cough of ≥2 weeks attending a large governmental referral centre in Sana'a, Yemen, between 2009 and 2010, were eligible to participate.

**Results:**

497 and 446 (89.7%) participants were surveyed the first and second day of attending the services and 48 IDIs and 12 FGDs were also conducted. The majority of patients were disadvantaged and had poor literacy (61% illiterate), had travelled from rural areas (47%) and attended with companions (84%). Key barriers for attendance identified were clinic and transport costs (augmented by companions), distance from home, a preference for private services, strong social stigma and a lack of understanding of the diagnostic process. There were discrepancies between patient- and doctor-reported diagnosis and 46% of patients were unaware that TB treatment is free. Females faced more difficulties to attend than men. The laboratory practice of providing first-day negative smear results and making referrals to the private sector also discouraged patients from returning. Strategies to bring TB diagnostic services closer to communities and address the multiple barriers patients face to attend, will be important to increase access to TB diagnosis and care.

## Introduction

Tuberculosis (TB) is one of the leading causes of adult morbidity and mortality [1] and the poorest sections of society carry the highest burden of disease [2]. Limited access to TB diagnosis is a barrier to accessing treatment and one third of the estimated new cases of TB are missed by National TB Programmes [3]. Increasing case detection is an international priority, however the obstacles to accessing services are complex and rooted in disadvantage and exclusion [4]. Obtaining a diagnosis of TB is a prerequisite for obtaining free treatment [5], yet diagnostic facilities are often sparsely distributed, particularly in rural settings, and access can be difficult [6] and costly [7]. The majority of individuals with TB live a precarious existence on a low income and are preoccupied with immediate survival [8]. Attendance can be delayed by misconceptions about disease causation and transmission [9], use of traditional [10] and private medicine [11], unfamiliarity with services [12], fear of prejudice [13] and the restricted liberties of women [14]. Alternatively, symptomatic adults simply do not reach formal services and remain in the community.

Yemen, the poorest of the Arab states, has a dispersed and predominantly rural population, a high incidence of TB [15] and a history of regional and national conflict. There are indications that patients face multiple obstacles to attend diagnostic services, incurring substantial expenses [7], sometimes abandoning the diagnostic process and failing to register for TB treatment [16], [17]; however there are no comprehensive studies of these barriers in Yemen. The health seeking behaviour of symptomatic adults prior to diagnosis, the reasons for delay and the considerable costs incurred have been well documented elsewhere [18]. Less well known are the experiences of adults that reach diagnostic services and the reasons for dropping out *during* diagnosis. This study therefore investigated economic, geographical, socio-cultural and health system factors hindering adults' attendance and completion of the TB diagnostic process and treatment registration, to inform interventions that will improve patient access to services.

## Methods

### Ethics statement

Ethical approval was obtained from the Liverpool School of Tropical Medicine Research Ethics Committee (Research Protocol 08.49) and Sana'a University. All participants were asked to provide informed consent to participate. For illiterate participants, the purpose of the study was explained, the information sheet read out and oral consent requested in the presence of a witness, who was asked to sign the informed consent form. All other participants were asked for written consent. Most patients in the study settings agreed to participate, but were suspicious of signing documents. Patients who wished to provide written consent, signed the forms. Any person reluctant to sign, was asked whether they agreed to participate by providing oral consent and if they agreed, the procedure for illiterate participants was followed. These procedures were specifically approved by the ethics committees.

The study was based at the National Tuberculosis Institute (NTI) in Sana'a; a large governmental referral centre on the city perimeter that provides TB diagnostic services to referred and self-referred patients. The Centre has a high turnover of patients from a wide geographical area; most with limited financial resources. The NTI serves as the reference centre for TB and has a prestigious reputation among the population. Although the National Programme has tried to decentralise services to peripheral diagnostic facilities, more than 90% of sputum examinations are conducted in this Centre. The Centre therefore receives a wide range of patients from across Sana'a and neighbouring governorates.

The study employed a mixed methods design comprising a cross-sectional survey and in-depth-interviews (IDIs) and Focus Group Discussions (FGDs). The survey was conducted on patients' arrival at the Centre and investigated the social networks supporting the patient and prior health seeking behaviour, knowledge and perception of TB and of the services. As part of the survey follow up, exit interviews completed with the same patients the next day assessed their experience, understanding of and attitude towards the diagnosis. The diagnosis reported by the patient was compared with the diagnosis recorded by staff. Questionnaires were based on experience of interviewing patients in Yemen and other countries, although it is recognised the instruments had not been formally validated [7], [19]. IDIs aimed to explore in detail the barriers and enablers to completing diagnosis and accessing treatment, the reasons for defaulting and to obtain suggestions for improving services. FGDs aimed to stimulate further discussion of the issues emerging from the IDIs.

### Participants

Adults with cough of ≥2 weeks attending the NTI from February 2009 were eligible to participate in all study components. Participants for the survey were selected using systematic random sampling from the daily patient register. A sampling interval was used to select 6 patients per day (30 per week) and to enrol a representative sample of patients spread out over the day until the sample size was reached.

Participants in qualitative studies were selected from a logbook of TB suspects, kept by the Centre as part of the research and routine monitoring processes. Patients were placed into 4 categories: those that had completed the diagnostic process; those that had failed to return in the succeeding days and had dropped out of the process; and smear positive patients that had registered or failed to register for treatment. Diagnostic default was defined as an individual not returning to the diagnostic centre after initial sputum submission. Patients could also default after receiving a smear-positive diagnosis, prior to initiating treatment [20]. Patients that had taken part in first or second day quantitative interviews were not included in the qualitative study to avoid overburdening them. Participants were selected purposively from each category to obtain a range of ages, representation of both sexes, cough duration, rural and urban residents from different geographical areas and immigrants.

Participants in the IDIs were individuals that had abandoned or had completed the diagnostic process. Patients abandoning the process were invited by phone, using the contact details obtained on enrolment. IDIs were conducted face-to-face at the patient's home, in discreet locations in the health centre or at another mutually convenient setting and followed semi-structured interview guides. Individual interviews with patients were difficult to obtain, as patients were reluctant to be separated from the family members or friend(s) accompanying them. Companions frequently participated in the interviews and this was noted by the research interviewers. Participants in FGDs were patients that had completed the diagnostic process and patients receiving treatment. The FGDs were conducted separately for male, female, older and younger participants. Discussions were held at locations convenient to the patient and patients were grouped geographically. Discussions and most interviews were conducted in Arabic and a local translator was used for other languages. Subsistence and transportation costs were offered to patients and relatives.

### Sample size

A sample size of 500 participants was calculated for the survey; assuming that some parameters of interest could have a prevalence of 50% and to attain a precision of +/−5%. Participant enrolment in the qualitative studies continued until little fresh information was obtained. Forty-seven IDIs and 12 FGDs were conducted. Of these, 16 IDIs and 2 FGDs were conducted among patients completing the diagnosis, 11 IDIs among participants abandoning the diagnosis and 20 IDIs and 10 FGDs among patients who had registered for treatment.

### Data management and analysis

Quantitative data were entered into *Epi-Info* and analysed using summary descriptive statistics. A thematic analysis of qualitative data was undertaken following the Framework approach [21]. Interview transcripts were organised using *NVivo 9.2* software and linked to patient demographics. The research group developed and fine-tuned a coding framework and collated data using a Framework Matrix. Researchers attended a workshop to discuss meaning, interpretation and key themes, providing the foundation for broader theoretical and conceptual development. The researchers differentiated between the perspectives of patients and their companions when interpreting and presenting the data. Views of patients completing, defaulting or registering for treatment are presented together and differences in viewpoint between the groups highlighted.

## Results

### Survey

A total of 497 participants were enrolled in the first day survey and 446 (89.7%) of these were re-interviewed on the second day of attendance. Participants had a mean (SD) age of 42.8 (18.4) years (Table 1), 251 (50.8%) were male, 232 (46.7%) resided in rural areas and 302 (60.9%) were illiterate. Only 34 (7%) were foreigners, mostly Somalis. Most participants had large households with a median of 8 residents. The majority of participants were accompanied (418, 84%; 95% CI = 80.6–87%), frequently by two or more companions. Most of these companions were relatives other than a spouse and less frequently friends, neighbours or work colleagues. Only 65 (25.9%; 95% CI = 20.9–31.7%) males and 12 (4.9%; 95% CI = 2.9–8.4%) females attended alone (p<0.001). Returning patients (N = 446) often received financial support or assistance-in-kind from a relative, including money (275, 65.7%), transport (276, 61.9%) and food (262, 58.7%). Partners were also important sources of support providing money (54, 12.1%), transport (49, 11%) and food (47, 10.5%) and, to a small extent, friends (3.8%, 4.5% and 3.6% respectively). Most patients (395, 80.9%) had made a median of two (range 0–20) visits to other health providers prior to attendance, particularly to private practitioners. Fewer had consulted traditional healers, obtained over-the-counter medicines or self-medicated, as shown in table 1.

**Table 1:** Characteristics of participants attending TB diagnostic services and their companions.

Participants		N = 497 (%)	95% CI
**Mean age (SD) [range]**		42.8 (18.4) [18–90]	
**Male (%): Female (%)**		251 (50.8): 243 (49.2)	(46.4–55.2)
**Residency**	Sana'a	247 (49.7)	(45.3–54.1)
	Rural	232 (46.7)	(42.3–51.1)
	Other town	18 (3.6)	(0.2–0.6)
**Education**	None	302 (60.9)	(56.4–64.9)
	Primary incomplete	77 (15.5)	(12.6–18.9)
	Primary complete	33 (6.7)	(4.7–9.2)
	Secondary or higher	84 (17.0)	(13.9–20.5)
**Sought help elsewhere**		395 (80.9)	(75.7–82.8)
**Median consultations [range]**		2 [0–20]	
**Service consulted**	Private practitioner	255 (62.2)	(46.9–55.7)
	Public hospital	177 (43.2)	(31.5–39.9)
	Pharmacy/over the counter	82 (20.3)	(13.5–20)
	Traditional healer	31 (7.7)	(4.4–8.7)
	Chest hospital	24 (6.0)	(3.3–7.1)
	Primary Health centre	21 (5.2)	(2.8–6.4)
	Self-treated	16 (4)	(2.0–5.2)
**Attended alone**	Males	65 (25.9)	(20.9–31.7)
	Females	12 (4.9)	(2.9–8.4)
**Attended with company**		418 (84.3)	(80.6–87.0)
**Number of companions**	1	212 (44.5)	(38.4–47.1)
	2	143 (30)	(25.0–32.9)
	3	54 (11.3)	(8.4–13.9)
	≥4	16 (3.3)	(2.0–5.2)
**Accompanying person**	Relative other than spouse	341 (83.6)	(79.7–86.9)
	Spouse/partner	49 (12)	(9.2–15.5)
	Friend	12 (2.9)	(1.7–5.1)
	Neighbour	6 (1.5)	(0.01–3.2)

#### Understanding and experience of diagnostic services

Many participants had been treated previously for TB (100, 20.1%; 95% CI = 16.8–23.9%). Most of the remaining 397 new patients arriving at the Centre had heard of TB (316, 79.6%), knew it was curable (297, 74.8%) and transmitted by another person (266, 67%) (Table 2). But many also believed transmission was associated with cold air (79.1%), animals (62.5%) and diarrhoea (39.3%), with some indicating TB could be inherited (20.2%). A high number of survey participants (214, 53.9%; 95% CI = 49.0–58.7%) were unaware TB treatment was free. Of 120 patients who reported having TB on exit, only 81 (67.5%; 95% CI = 58.7–75.2%) had this diagnosis recorded in the clinic files (Table 3). Conversely, 9 of 264 (3%; 95% CI = 2–6.4%) patients who said they did not have TB, were recorded as having TB. Most participants were satisfied with diagnostic services (399, 80.3%), the attitude of staff (422, 84.9%) and information received (360, 72.4%). Patients felt they could ask questions (418, 84.1%), however, 70 (14.1%) were unsure of their diagnosis.

**Table 2:** Knowledge of TB among new patients (Questionnaire 1).

	N = 397, 79.9% (%)		95% CI
**Had heard of TB**		316 (79.6)	(75.4–83.3)
**Correctly responded TB**	Can be treated	297 (74.8)	(70.3–78.8)
	Is linked to HIV	40 (10)	(7.5–13.4)
	Is acquired from a patient with TB	266 (67)	(62.2–71.4)
	Can be prevented	298 (75.1)	(70.6–79.1)
	Treatment is free	188 (47.4)	(42.5–52.3)
**Incorrectly responded TB**	Is inherited	80 (20.2)	(16.5–24.4)
	Can be caught from diarrhoea	156 (39.3)	(34.6–44.2)
	Can be caught from cold	314 (79.1)	(74.8–82.8)
	Can be caught from animals	248 (62.5)	(57.6–67.1)

**Table 3:** Agreement between the diagnosis reported by the patient and the doctor (Questionnaire 2).

	Patient understanding of diagnosis			
Medical diagnosis	TB (%)	Not TB (%)	Not recorded (%)	All (%)
**TB**	81 (67.5)	9 (3.4)	4 (5.6)	94 (20.6)
**Not TB**	39 (32.5)	255 (96.6)	68 (94.4)	362 (79.4)
**All**	120	264	72	456*

*Information was not collected in 41 patients.

#### Disclosure

Most first-day participants indicated they intended to disclose their diagnosis (404, 81.3%), especially to relatives (92.6%) or spouses (72.7%). Very few intended to inform employers (12.4%), work colleagues (20.2%), religious or community leaders (8.6%).

#### Women's access

Most first-day participants indicated that women had the same access to healthcare as men (419 of 497; 84.3%; 95% CI = 80.9–87.2%) (Table 4). Nevertheless, it was acknowledged that in practice women faced additional difficulties to attend the services (278, 55.9%; 95% CI = 51.6–60.2%), including: having to request permission from their husband (275, 55%; 95% CI = 50.9–59.6); being unable to travel alone (276, 55.5%; 95% CI = 51.1–59.8%) or to access household funds (71, 14.3%; 95% CI = 11.5–17.6%) and holding caring responsibilities (111; 22.3%; 95% CI = 18.9–26.2%).

**Table 4:** Reasons why women face difficulties accessing healthcare facilities (Questionnaire 1).

Believe that women	N = 497 (%)	95% CI
**Have same access to healthcare as men**	419 (84.3)	(80.9–87.2)
**Face difficulties accessing healthcare**	278 (55.9)	(51.6–60.2)
Need to ask permission of husband	275 (55)	(50.9–59.6)
Cannot travel alone to health services	276 (55.5)	(51.1–59.8)
Need to care for other relatives	111 (22.3)	(18.9–26.2)
Cannot use household funds	71 (14.3)	(11.5–17.6)

### IDIs and FGDs

Participants in IDIs included 16 adults who had completed and 11 who had abandoned the diagnostic process, and 20 who had registered for treatment. Participants completing diagnosis comprised 6 females and 10 males, aged 22 to 60 years, of whom 13 were married. Seven participants resided outside Sana'a. Three females and 5 males were working and 4 participants had schooling. Participants abandoning the diagnosis comprised 3 females and 8 males, aged 20 to 83 years, of whom 6 were married. One participant resided outside Sana'a. None of the females and 5 males were working and 8 participants had schooling. Participants who had registered for treatment comprised 13 females and 7 males, aged 18 to 50 years.

The FGDs with patients completing the diagnosis and patients registering for treatment were conducted separately for males and females and included urban and rural residents and Somalis.

#### Support and companionship

The whole family was often involved in accompanying the patient, providing financial support, transport, accommodation, completing paperwork, collecting medications or assisting with childcare and household tasks (quotation 1 (Q1)). Patients were encouraged to attend and received advice on healthcare seeking and taking the medications (Q2), although patients not completing diagnosis reported fewer supporting psychosocial factors. Nevertheless, many patients felt unsupported (Q3) and Somali refugees in particular reported hunger, isolation and language barriers.

Q1 A female patient attended the centre with her mother, brother and a friend.

Patient: *“My mother* [is my main supporter]. […] *My brothers [supported me] financially and morally.”* […]

Patient's brother: *“It's a long journey* [from Al-Hada]. *We have to change more than once.* […] *It takes two and half hours* […] [by taxi]. [It cost] *1500 each. We are staying at a friend's.”*

(Female, completed diagnosis, IDI)

Q2 *“to be honest brother*, […] *the moral support is more important than the financial support”*.

(Male, not completing diagnosis, IDI)

Q3 *“My husband was not bothered. He told me: ‘It is up to you if you want to go or not’.* […] *Actually, I was concerned about my unborn baby and my wellbeing and the children's. My husband used to say: ‘Nothing wrong with you’”.*

(Female, not completing diagnosis, IDI)

#### Health-seeking behaviour

Patients made several visits to the same provider before attending the Centre and moved between public and private providers during and after attendance for diagnosis. Private healthcare was the preferred choice (Q1). Individuals who had not completed their screening often chose, or were referred to, private practices (Q2, 3).

Q1 Patient's son: “[My mother] *means those who can afford it go to private hospitals.* […] *Those with limited resources come to this centre.”* […]

(Female, completed diagnosis, IDI)

Q2 Patient: *“They said: ‘Come the next day’.* I [should have] *had a second saliva test. One before breakfast and one after breakfast. But I did not come back.* […] *I went to another* [private] *doctor and he told me that I am not having that disease* [TB]”

(Female, not completing diagnosis, IDI)

Q3 Patient: *“I was not asked* [to come back again]. *Only the doctor asked me to visit his* [private] *clinic* [near my home]”.

(Female, not completing diagnosis, IDI)

#### Knowledge and understanding

Many patients knew of someone with TB and on arrival were aware of measures to prevent cross-infection. Several believed they had contracted TB from a relative. Many patients were unclear of the rationale for the repeated sputum examinations (Q1) and doubted or misunderstood their diagnosis (Q2). Patients were generally uninformed about diagnostic services in a nearer governorate and the availability of treatment closer to home and many preferred to bypass them.

Q1 Patient: “[…] *the second day test was not needed and the doctor could have done it on the first day. He was free. He was FREE! He had nothing to do.”*

(Male, completing diagnosis, IDI)

Q2 Patient with smear negative TB receiving treatment at the Centre: “*I was told that I have chest infection only.* […] *When I came here I showed them the reports I brought with me from the* […] *hospital. I was told to do a phlegm test. The first was fine, the second was also fine - so was the third.* […] *Praise to Allah, it showed negative* […]. *The doctor asked me questions and told me that I had fever and a cold.”*

(Male, registered for treatment, IDI)

#### Experience of diagnostic services

Some patients perceived the Centre to be efficient and appreciated its specialism in TB. However, others found the Centre overcrowded, disorganised and unhygienic, staffing inadequate and unprofessional (Q1, 2). Concerns were expressed about service disruption for power failures, holiday closures and restricted opening hours. Patients disliked being referred out to private laboratories. Doctors were perceived to be busy and consultations were considered rushed, intimidating and lacking privacy. Few patients felt they had received adequate health information.

Q1 Patient's son: *“There is a shortage in the doctors in this centre. I have seen some patients suffering on the ground and there's no doctor to see them. Some get upset and leave the centre without being seen* […]*!”*

(Male, completed diagnosis, IDI)

Q2 *“*[…] *when I come in at the Centre's door, I do not feel that I am in a hospital; I feel like being in a market.* […] *The rooms, the people there… management wise there is no order.* […] *you do not feel that you are in a hospital which* [is] *supposed to be fighting a very dangerous disease.* […] *You feel as if you are in a market and going from one shop to another; crowds all over the place.* […]*”*

(Male, not completing diagnosis, IDI)

#### Disclosure

Patients made very different decisions about disclosing their diagnosis. Some intended to inform only selected members of the household and others neighbours, work or study colleagues. Others had anticipated negative repercussions and had moved to the city to avoid stigma or withheld their diagnosis at work/college. Some patients reported their diagnosis had damaged relationships with family members, neighbours or work colleagues, leading to marginalisation or rejection and loss of employment or income (Q1,2). Others, however, believed they had a duty to be transparent, regardless of any possible negative reaction.

Q1 *“The social effects sometimes are worse than the illness itself* […]. [Yemen is] *a very illiterate community* [with little awareness of the disease]*”*.

(Male, completing diagnosis, FGD)

Q2 Female patients, completing diagnosis, FGD:

Participant 1: *“Some patients get scared about the disease. They fear to face the truth and prefer to stay at home without seeking medication.”* […]

Participant 3: *“Some are concerned about their safety. Even my brothers stopped visiting me.”*

Participant 4: *“If I happened to cough at my* [work] *colleagues, they would step back from me and then I was told to stay home until I get well”*

#### Costs

Patients felt the direct and indirect costs of diagnosis and treatment were onerous and some had borrowed money. The cost of transportation, private tests, loss of earnings, prescriptions for non TB medication and clinic fees were most important (Q1). Despite clinic fees being an important cost component, some considered them reasonable compared with private sector fees (Q2). Patients with a diagnosis other than TB did not always understand why they were charged for treatment, when patients with TB received free treatment. Some patients decided not to collect prescriptions (Q3).

The charges reported for the services provided varied considerably and sometimes over the standard rate, suggesting instances of overcharging. Some patients also confided they had been asked for extra payments in return for faster examinations and test results. Few patients reported paying for food and accommodation, commonly staying with relatives or friends. Patients receiving TB treatment indicated that collecting the drugs caused financial strain and often negotiated with staff to attend less frequently.

Q1 Patient's father: “[…] *We can't afford all this money for transport and medication.* […] *The tests and medication had to be obtained from outside the centre and that is expensive.* […] *The blood test cost me YER 2,000 and the medication YER 4,500. That is too much!* […] *It is not easy to go every day and to buy from private places. If you give the medication in the Centre, the transport cost is easy to afford.”*

(Male, not completing diagnosis, IDI)

Q2 *“Your charges are really good and cheap - even the poor can afford it. For example in [name] hospital, the X-ray costs YER 1,400, but in your centre it is really cheap - just YER 50.* […] *That's only regarding the tests, like the saliva test and the X-ray, but I have no idea about the* [cost of] *medication.”*

(Female, not completing diagnosis, IDI)

Q3 A female patient did not purchase the prescribed medication:

“[…] *I told the doctor: ‘I have to go for my prayers now. I will buy it* [the medication] *later.’, but I did not.”*

(Female, completed diagnosis, IDI)

#### Women's access

Although patients did not speak easily about gender differences, females were considered to lack autonomy to travel (Q1), access household finances, make healthcare decisions and leave household duties. Women sometimes presented late (Q1) and pregnant women found travel especially difficult. Patients expected to be examined by same sex staff (Q2) and some men acknowledged they would not necessarily seek healthcare for female relatives (Q3).

Q1 Patient: *“The lady* [at the Centre] *asked me: ‘Why were you late to come up, if you had the cough for long?’ I told* [her because of] *the transport and my brother is always busy. And I can't go there alone. I have to have someone with me. My brother for example.”*

(Female, not completing diagnosis, IDI)

Q2 *“And the X-ray room for the chest test, there should be two rooms; one for the females and one for the males. The staff who did the X-ray for me was a male and I was very embarrassed to be honest. If it was a female staff it would have been better.”*

(Female, not completing diagnosis, IDI)

Q3 *“*[…] *some* [men] *do not even believe in medicine and if their sons tell their dads about the importance of the hospitals, they just say: ‘No, let her* [the mother] *die at home’.”*

(Male, completing diagnosis, FGD)

#### Returning for a second day

Many patients experienced difficulties to return the second day, citing financial constraints (including lost revenue), distance (Q1), the need for accommodation, poor health, responsibilities at home and work (Q2), and barriers for women. Patients defaulting diagnosis felt they did not have to return the second day if the first day test results had been revealed and were said to be negative, or if the information received from the staff suggested they did not have TB (Q2).

Q1 *“*[…] *if I could afford it I would have returned; but it is too expensive. Can you imagine it costs YER 1,000 to go to the Centre? It's a long distance and costs a lot.* […] *If I could afford it I would go, because my health is more important* […]*”*

(Female, not completing diagnosis, IDI)

Q2 *“I was not asked to go back, but only heard from some* [other] *patients*. […] [Also, I did not come back because of] *my commitments and work. Plus the doctor told me: ‘Do not worry you are negative. It is only mild infection’.* […] *I was relieved when the doctor told me: ‘You are negative’.”*

(Male, not completing diagnosis, IDI)

#### Enabling factors

Factors facilitating completion of diagnosis included geographical proximity, having adequate funds, a private vehicle, a supportive network of family and friends, and employment rights. A key driver for attendance was a belief in the importance of individual and family health.

#### Improvements

Patients suggested improving service delivery by employing additional staff, extending opening hours, segregating X-ray facilities and performing blood tests. Improvements to the infrastructure included the provision of hospital beds, better waiting areas, a CT scanner, computerised records, a pharmacy and green space (Q1). Social assistance patients would value included accommodation and food for patients and a shuttle transport service (Q1). Changes to practice encompassed improved infection control and information-giving, encouragement from doctors, follow-up calls, and ending unofficial payments and preferential treatment (Q2). Patients emphasised the need to raise awareness of TB, the Centre and services available outside Sana'a. Patients wanted the diagnosis to be completed in a day, additional centres, decentralised diagnostic, retesting and treatment services and a mobile clinic (Q2, 3).

Q1 Patient's brother: *“If you offer better facilities in the countryside, that would be much better and cheaper - especially* [for] *those with less money. Provide specialist doctors. We have some clinics, but no doctors available.”*

(Female, completed diagnosis, IDI)

Q2 Patient's husband: “[The charge is reasonable] *compared with the private hospitals [but] I met some patients who are really poor and they can't afford to pay the YER 200* [for registration]. *I suggest there should be a charity organisation or some officials to pay for them. […] I saw a very poor lady outside.* […] [Otherwise] *we think that if you can provide wards and beds at the hospital for those extremely ill patients that will be very good.”*

(Female, completed diagnosis, IDI)

Q3 Patient's husband: [You could help patients to come back the next day to complete the tests] *by encouraging them. When you encourage them and comfort him* [the patient] *by telling him it is curable and make things easy, that will make him come back. And refer him to local centres to collect medication.* […] *Give him enough information about the illness. The doctor can do the role of the father and a mother and make the patient* [feel] *comfortable.”*

(Female, completed diagnosis, IDI)

## Discussion

Our study documents that the majority of patients in Yemen struggle to reach the Centre, find it difficult to return for further visits and have little understanding of the importance of completing diagnostic tests. Patients were typically poor, attended with company and had travelled. Females faced particular adversity. Like migrant populations elsewhere, the Somali refugees in Sana'a appeared to be particularly susceptible to contracting TB and to experiencing difficulties in accessing diagnosis and treatment, due to high mobility, marginalisation and low socioeconomic status [22]. Despite making considerable sacrifices, several patients failed to complete the diagnostic process. Competing pressures at work and home jeopardised completion and were sometimes compounded by a negative experience at the diagnostic centre. The practice of supplying first-day smear results and referrals to the private sector discouraged return. Survey participants expressed a high level of satisfaction concerning their experience, while IDIs and FGDs provided a platform for participants to convey criticism and reveal a plethora of obstacles.

The demographic profile of patients completing diagnosis did not appear to differ greatly from patients abandoning the diagnostic process, nor did their experience of diagnostic services. The majority of patients faced multiple barriers to attend, many of which are markers of multi-dimensional poverty [23]. Often adverse circumstances combined to prevent a patient from staying the course.

### Socio-cultural and health system barriers

Consulting other healthcare providers before formal TB diagnostic services has been widely reported and patients in Yemen often made several visits to the *same* provider - a known risk factor for diagnostic delay [24]. Use of private healthcare before, during and after attendance at diagnostic services, has also been reported in other settings [25]. The low awareness of free treatment among patients cannot be assumed to have acted as a deterrent for attendance. Free treatment can be an incentive or a deterrent, according to public perception of the quality of free public programmes and individual ability to pay for private care [12]. The misconceptions about disease transmission held by many patients are common [26], often affecting public behaviour and eventually increasing transmission [27]. The considerable disagreement between the diagnosis reported by patients and staff might stem from genuine lack of understanding, self-denial or fear of stigma among patients with TB, or false claims of TB in the hope of remuneration. Whatever the cause, our findings highlight the need for clear and simple communication by health staff and demonstrate that information obtained from patients at exit interview can be unreliable.

Most patients (and particularly women) attended services with several companions. Although companions often provided material, practical and psychosocial support, this was counterbalanced at times by their contribution to delayed attendance, inflated costs and the promulgation of prejudice and misinformation.

TB is associated with stigma [28]; the severity, causes and manifestations of which vary across cultures [29]. Patients and their families may withdraw socially and experience rejection, divorce, reduced marriage prospects and dismissal from school or employment and Yemeni patients were fearful of the social consequences of the disease. Women are particularly vulnerable to rejection by a spouse or fiancé in societies in which they are economically dependent [30] and this is likely in Yemen. Yemen has one of the lowest rankings of gender parity in the world [31]. The North is characterised by a traditional tribal and conservative culture and it is likely many women are unable to attend. Equitable access is vital for successful TB control and diagnostic services need to accommodate the differential needs of females to access services [32]. Patients' reluctance to disclose their diagnosis could be emblematic of perceived or enacted stigma [33]. Unfortunately, failure to disclose diagnosis precludes the possibility of obtaining social support from family or peers, which is a key contributor to treatment adherence [34]. Some patients were isolated and patients defaulting diagnosis reported lower levels of financial or employer support.

### Economic and geographical barriers

Although Yemen has a decentralised DOTS service [35], most patients considered the location of services too distant for diagnosis and collection of medicines. Public transportation in Yemen is expensive and does not always extend to mountainous areas. Rural residency impedes patient attendance at diagnostic services [19] and a high proportion of patients were referred, or self-referred, to the reference centre from across the country, travelling considerable distances. The financial impact of transport, clinic and private laboratory fees, coupled with loss of earnings, was considerable [36] and many patients had borrowed money [37], as reported in other settings. The presence of companions multiplied these costs, particularly for women, who were nearly always accompanied. Although seldom reported, an important cost component were the additional payments requested by staff [38] and the cost of diagnosis was also increased by frequent referral to private services for additional tests. Many patients experienced a reduction in income due to illness, as reported by others [39]. Economic and geographical barriers to patient attendance and completion of diagnosis reaffirm the need to locate diagnostic and treatment centres close to the community [40], especially in countries with difficult terrain and multiple barriers for displacement, such as Yemen. In addition, clinic charges impede access to diagnosis and making services free could improve attendance [41].

### Methodology and study limitations

Few studies employing mixed methods have been conducted in Yemen and this is the first to elicit patients' opinions of health services. The IDIs and FGDs added insights into the patient experience that were not apparent in the survey and succeeded in eliciting the views of defaulters. We were unable to interview patients not registering for treatment; however patient adherence improved due to study effect and only a few patients defaulted diagnosis or did not register for treatment. Obtaining the unique perspective of the patient (and in particular females) was difficult, as a more senior person, or male accompanying a female patient, would tend to answer on behalf of the patient. It is feasible that this situation compromised patient confidentiality and the validity of responses on occasions, especially regarding sensitive topics, such as a woman's freedom to seek healthcare. It may also have contributed to the lack of an obvious distinction between women's and men's experiences of attending services. Political instability and cultural norms for female interviewers restricted travel and meant that IDIs and FGDs could only be held in Sana'a governorate. There were therefore few possibilities to obtain the perspective of patients who resided in other towns, after they had left the Centre.

### Conclusions

Many factors hinder patients' attendance at diagnostic services. This study described the experiences of patients who reached TB diagnostic services; however there is increasing evidence that many symptomatic adults remain in the community. It is probable that these adults have fewer social and economic resources, less education and contain an overrepresentation of females. Strategies to bring diagnostic services closer to communities and address the multiple barriers patients face will be important to increase access to tuberculosis diagnosis and care. Access barriers to TB diagnostic and treatment services are likely to have been exacerbated by the political conflict that affected Sana'a after completion of fieldwork.
